# How variation in head pitch could affect image matching algorithms for ant navigation

**DOI:** 10.1007/s00359-015-1005-8

**Published:** 2015-04-21

**Authors:** Paul Ardin, Michael Mangan, Antoine Wystrach, Barbara Webb

**Affiliations:** School of Informatics, University of Edinburgh, 10 Crichton St, Edinburgh, EH8 9AB UK

**Keywords:** Navigation, Route following, Ant, Pitch, Retinopic matching

## Abstract

Desert ants are a model system for animal navigation, using visual memory to follow long routes across both sparse and cluttered environments. Most accounts of this behaviour assume retinotopic image matching, e.g. recovering heading direction by finding a minimum in the image difference function as the viewpoint rotates. But most models neglect the potential image distortion that could result from unstable head motion. We report that for ants running across a short section of natural substrate, the head pitch varies substantially: by over 20 degrees with no load; and 60 degrees when carrying a large food item. There is no evidence of head stabilisation. Using a realistic simulation of the ant’s visual world, we demonstrate that this range of head pitch significantly degrades image matching. The effect of pitch variation can be ameliorated by a memory bank of densely sampled along a route so that an image sufficiently similar in pitch and location is available for comparison. However, with large pitch disturbance, inappropriate memories sampled at distant locations are often recalled and navigation along a route can be adversely affected. Ignoring images obtained at extreme pitches, or averaging images over several pitches, does not significantly improve performance.

**Electronic supplementary material:**

The online version of this article (doi:10.1007/s00359-015-1005-8) contains supplementary material, which is available to authorized users.

## Introduction

The field of neuroethology owes a great debt to the pioneering work of Prof Rüdiger Wehner. Of particular note are his studies of navigation in desert ants, which have been fundamental in revealing how a ‘toolbox’ of simple mechanisms gives rise to complex guidance behaviour (see Wehner 2008 for review). His work also laid foundations for the emerging fields of computational biology and biorobotics by providing benchmark behavioural assays against which functional hypotheses, embodied as computer programmes, can be verified.

An example of his lasting legacy is found in the observation of visual homing behaviours in desert ants (Wehner and Räber 1979; also reported in bees by Cartwright and Collett 1982, 1983), which inspired the long-standing hypothesis that insects store visual memories retinotopically, allowing the animal to return to a location by moving so as to increase the retinotopic match between what they currently see and their memory. This concept inspired a family of computational models able to reproduce visual homing behaviour in various experimental scenarios (Cartwright and Collett 1983; Hafner 2001; Möller 2001; Stürzl and Mallot 2006; Vardy and Möller 2005; Zeil et al. 2003). The same retinotopic principle has been in used in the ‘visual compass’ or ‘alignment image matching’ hypothesis for recovery of heading direction (Zeil et al. 2003; Collett et al. 2013), i.e. that the heading direction when an image was stored can be recovered by physically rotating to find the minimum in the image difference. Coupled with dense storage of multiple images, this has recently been shown to be an effective method (Baddeley et al. 2012) for recapitulating routes in complex environments as observed in ants (Kohler and Wehner 2005; Mangan and Webb 2012; Wehner et al. 1996). The scanning behaviour that has been described in ants (Wystrach et al. 2014) is also consistent with the assumption that they are attempting to find a retinotopic match, rather than being able to recognise a rotated image.

These algorithms have been shown to work in simulation, and in some cases on robots, but are not always challenged with realistic stimuli as experienced by the ant in its natural habitat. For example, in our own field studies of *Cataglyphis velox* following routes through scrubby undergrowth, the highly cluttered environment lacks any distinctive or consistently visible landmarks (Mangan and Webb 2012). Algorithms are often tested with higher resolution than the eye of an ant (inter-ommatidial angle around 4 degrees), or with complete omnidirectional views, whereas the ant has a significant rear blindspot (Schwarz et al. 2011; Zollikofer et al. 1995). Additionally, few of these tests take into account the potential noise or alteration of the view due to the ant’s movement over uneven terrain, which might invalidate the retinotopy-based methods outlined above. To quote Wehner ‘… the snapshot is fixed relative to retinal coordinates and does not rotate within the ant’s head to compensate for changes in the orientation of the animal’s longitudinal body axis… This has important implications. If the snapshot is retinotopically fixed, and if it should later be matched to a current retinal image, this match can be accomplished only if the animal assumes the same orientation of its body as it did while acquiring the snapshot’ (Wehner et al. 1996). Note that this requires ‘the same orientation’ of the head in all three axes: yaw, pitch and roll.

If moving on uneven terrain causes the ant’s head to experience pitch and roll, this could affect the projection of the view onto the retina and hence the crucial information available about yaw orientation. Early reports of head stabilisation in navigating ants (Duelli 1975; Wehner and Räber 1979; Wehner et al. 1992) lack detailed analysis. More recent data suggest that even when walking on flat terrain the ant’s gait does not keep the head completely level (Reinhardt and Blickhan 2014). The latter result is for the wood ant *Formica polyctena*, but a similar range of movement can be measured for the desert ant *Cataglyphis fortis* running on flat ground (Steck et al. 2010; supplementary video). Desert ants inhabiting the extremely flat salt pans of North Africa might experience little additional substrate-induced disturbance, but many ants use visual memory in habitats with more uneven terrain, or even extreme conditions such as rough undergrowth and rainforest (Harrison et al. 1989), where walking up, down and along vegetation is necessary and will strongly affect posture. Ants have also been reported to successfully home (although possibly using path integration alone) when amputation of two legs leads to continuous stumbling (Steck et al. 2009). Head pitch is also influenced by the need to balance the load being carried (Moll et al. 2010); evidence that the posture is altered by the mass of a load is given in (Zollikofer et al. 1995) although the direct effect on head angle was not measured. Other studies have suggested that pitch compensation when walking on slopes is also far from complete, with a change in slope from −75 to +75 degrees inducing a change in the average caput-substrate angle of less than 40 degrees in *C. fortis* (Weihmann and Blickhan 2009). It is similarly reported in (Wohlgemuth et al. 2002) that ‘during ascent and descent walks (for slopes of 54 or 24 degrees) the ants had their heads inclined (relative to the skylight pattern) at an angle that differed from the one kept on even ground’ (p. 277).

These latter two studies show that the average head pitch relative to gravity or the horizon is substantially affected by the substrate for large-scale terrain features, but not the extent to which rapid variation in pitch might be induced by an uneven ground surface combined with the ant’s gait, or whether the ant stabilises its head at this time scale (note Steck et al. 2009 find that regular corrugations in terrain at 12- to 25-mm spacing are not compensated by gait adjustment). In this paper, we observed variance in head pitch using high-speed close-up videos of ants running, with different loads, over natural terrain. As we report, there is substantial variation and little evidence of stabilisation. We then use realistic modelling, with ‘ant-eye’ filtering of images taken from a 3D reconstruction of our field site, to assess whether the observed degree of pitch variation would have a significant effect on the ability of ants to recover heading direction from image matching. We also assess the effect of the induced error on the ability to follow routes through cluttered natural environments, under the simplifying assumption that the ant’s storage or matching of images is not directly linked to knowledge of its current head pitch. We revisit this assumption, and consider alternatives, in the discussion.

## Materials and methods

### Head pitch analysis

At a field site on the outskirts of Sevilla, Spain (37°20N, 5°59W), ants of the species *Cataglyphis velox* were trained to a trap feeder positioned approximately 2 m from the nest, where they were provided with cookie crumbs of various sizes. The direct route back to the nest was through a channel made by embedding two white plastic boards approximately 10 cm apart in the ground. Consequently, the ants were still running over their natural (earth and gravel) terrain, but would reliably pass a specific point for close-up filming, approximately half way through their homing run. A small section was cut out of one side of the channel wall and replaced with clear Perspex to give a side view of the animals. Due to its absorption of ultraviolet light, the Perspex would appear opaque to the ants and we did not observe any significant deviation of ants within the channel when passing this section. Ants were filmed using a high-speed camera (Casio EX-F1) at 300 fps with a macro lens. The field of view covered approximately 4 cm of the ant’s path.

**Fig. 1:**
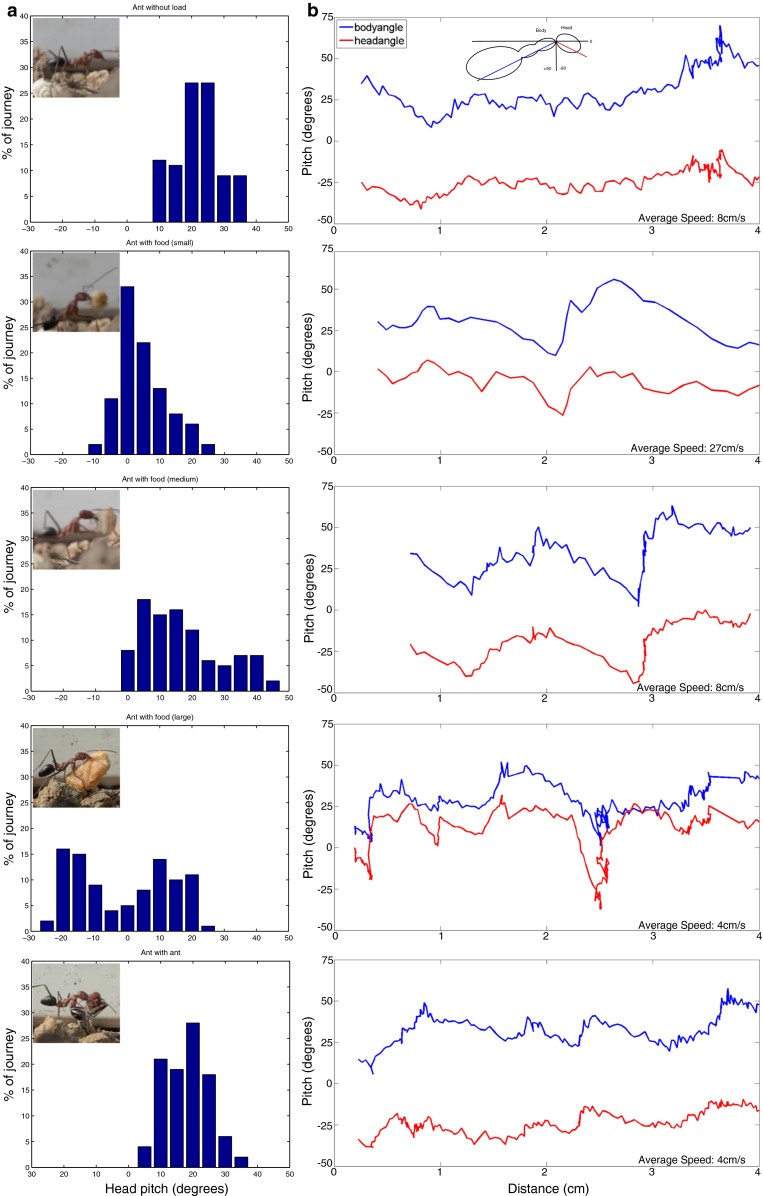
Head movement in navigating ants. Ants were recorded walking across the normal substrate at the field site in Sevilla, under 5 load conditions (from *top* to *bottom row*): no load, small food item, medium food item, large food item and carrying another ant as shown by the images *inserts*. **a** The distribution of head angles over the entire recording. **b** The instantaneous head and body angle of the ant as it moved across the camera field of view (*blue line*—body angle and *red line*—head angle). Angle conventions used are shown by the *insert*. The head of the ant moves across a substantial pitch range and varies with load. The attitude of the head is only partially decoupled from the motion of the body indicating that there is no continuous stabilisation of gaze

Five videos were selected for analysis on the basis of video quality and variety of items being carried by the ants (see Fig. 1). The head and body pitch was measured by analysing the videos with a custom programme developed in MATLAB (Mathworks Inc, USA). For each frame where the complete head and body was visible, the centre of the mandible and the dorsal joint of the head and neck were manually labelled and the resulting angle with respect to the horizontal image frame was computed. Similarly, the body angle was measured from the dorsal joint of the neck to the ventral joint of the alitrunk and petiole. We define the angle relative to the horizontal such that 90° points downwards.

### The simulated ant world

To test the effect of head pitch on the views experienced by ants, and the possible consequences for navigation, we created test scenarios in a 3D reconstruction of a natural environment, based on data from our study of route following in *Cataglyphis velox* (Mangan and Webb 2012). The field site used in that study was a flat semi-arid area covered in low scrub and grass tussocks. We mapped the tussock location and size and used panoramic pictures taken from ground level to estimate tussock height. From this, we generated a corresponding virtual environment, consisting of a 10 × 10 m area in which each tussock is represented as a collection of triangular grass blades of appropriate size and height, with a distribution of shading taken randomly from the intensity range in the panoramic pictures (Mangan 2011; Mangan et al. in preparation). Ground and sky have uniform intensities that differ from the grass blades. Within this virtual environment, the view from any position on the ground plane, facing in any direction, can be captured by a virtual panoramic camera. In particular, we can reconstruct the series of views along a path in this virtual environment that corresponds to the actual path we recorded for an ant in the real environment. The 3D world and image creation software are available at http://www.insectvision.org/.

**Fig. 2:**
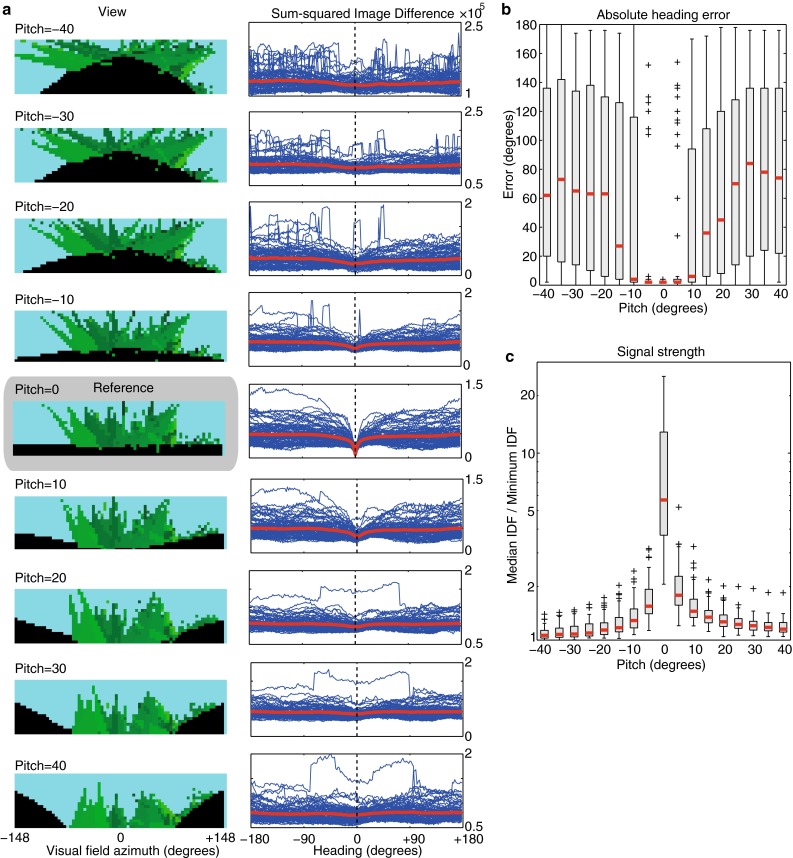
The effect of pitch on the visual compass. **a** *Left column* shows an example of the panoramic views generated at the same location in our 3D world but with pitch angle varying from −40° to 40°, introducing substantial distortion of the view. **a** *Right column* shows the rIDFs calculated at 81 test when comparing the reference image (no pitch) to the same image at differing pitch angles (−40° to 40°). *Blue lines* are the individual rIDF values for each location (aligned so the correct direction is at 0°), while the *red line* shows the mean across all the locations tested. At 0° pitch, the reference heading (0°) is readily identified as a minimum, but as pitch increases it becomes increasingly difficult to reliably extract the correct heading. **b** Range of heading errors computed across 81 test locations under varying pitch (median are shown in *red*, and inter-quartile range by *box*). Error increases significantly when pitch exceeds ±15°. **c** Signal strength, i.e. the median rIDF value divided by the minimum rIDF, as pitch varies. The depth of the minimum, and hence its detectability, drops dramatically with head pitch

A simulated ant eye was used to recreate the approximate retinotopic input that would be experienced by an ant. The eye model reproduces the projection of the visual world onto a uniformly curved retina (a spherical section) with a field of view (window in the sphere) that extends 296 degrees horizontally, and 76 degrees vertically, with 4 degree resolution. At zero pitch, the field of view is aligned such that the horizon runs through the centre of the image. Changing pitch rotates the field of view around the corresponding axis, so that the horizon line is changed and distorted as shown in Fig. 2a.

We used this virtual world in three ways in the following analysis.

- We first tested how, at any specific location, the ability of an ant to recover a heading direction using the visual compass method (see below) would be affected by the visual distortions produced by systematic alteration of the pitch of the head. This analysis was repeated for 81 different locations, which were taken at 10 cm intervals along a path corresponding to that of a real ant.
- We then used the distribution of pitches actually observed in the ant to investigate the directional error induced by pitch variation between the storing of visual memories and their use on a subsequent path traversal, under several different assumptions about how memories are stored and retrieved.
- Finally, to test whether the traversal of a whole route using repeated heading corrections is robust to the level of directional error caused by pitch variation, we test a simulated ant for its ability to follow the same route as a real ant under realistic levels of pitch variation.

Each of these analyses is now described in more detail.

### Visual compass and the systematic effect of pitch

The visual compass method recovers a heading direction by calculating the difference between a reference image and the images obtained as the viewpoint is rotated around the yaw axis. The difference is typically calculated as the pixel-wise sum square intensity difference between the images, that is:

$${\text{Image}}\;{\text{difference}} = \sum\limits_{i} {(I_{i} (x) - I_{i}^{r} )^{2} }$$

The change in this value as the viewpoint is rotated is called the rotational image difference function (rIDF). The minimum in the rIDF, i.e. the best match to the reference, should occur when the viewing direction is the same as that of the reference image. The directional minimum is generally robust to small displacements in location or changes in lighting etc. We tested whether it was robust to changes in pitch by calculating 360 degree rIDFs between a 0 pitch reference image and images generated at a pitch of −40, −20, −10, −5, 0, 5, 10, 20 or 40 degrees. This was repeated for 81 different images, which were generated in the simulated environment by taking images at 10-cm spaced locations along a route corresponding to that of a real ant.

We assess the effect of pitch firstly by directly visualising the rIDFs (Fig. 2a) and also using two measures of how distortion in the rIDF introduced by pitch could affect its use as a visual compass. The first measure is the absolute difference between the minimum in the rIDF, which a visual compass would select as the heading direction, and the actual heading direction for which the reference image was stored (Fig. 2b). We compare the median and interquartile range (IQR) of this error, for the 81 images, across different degrees of pitch alteration between the reference and test image. Second, if the rIDF is distorted (e.g. becomes noisier or flatter) due to pitch variation, detecting any minima could become more difficult. As an index of detectability, we use the ratio of the median rIDF to the minimum rIDF; if this is near 1, then the directional information available in the rIDF is low (Fig. 2c).

### Modelling variable head pitch of the ant during learning and retracing of routes

For an ant to use the visual compass to follow a route, it needs to learn images along the route, and then on subsequent traversal, compare what it currently sees to the stored images to find the heading direction that corresponds to the minimum in the rIDF. In the following, we store an image every 1 cm along an 8.12 m route and use this memory to recover a heading direction. The absolute error between the selected heading and the correct direction along the route is used as performance metric and is calculated for 81 locations spaced at 10-cm intervals along the route. To avoid distortions caused by perfect matches, we assume a minimum 1 cm difference between the test location and the nearest stored location.

As discussed in the introduction, previous tests of this type of algorithm have assumed zero variation in pitch, in either learning or testing, so we include this condition (zero pitch) as a control. We compare this to the following more realistic situations:

*Small pitch* The stored images along a route are each pitched by a value randomly sampled from those observed for the ant carrying a small cookie (Fig. 1a, second row), with the mean shifted to 0º resulting in pitch values ranging from −20.6º to 12.99º. At test locations, the pitch is randomly varied using the same distribution. Note that this assumes that the variation in the pitch is due to the close interaction of the ant’s stepping pattern and small-scale terrain rugosity, and hence, there is no significant correlation of the pitch between learning and test runs.

*Large pitch* More extreme pitch angles are simulated by randomly sampling from the pitch angles observed for the ant carrying a large cookie (Fig. 1a, fourth row), again following mean shifting to 0º. Head pitch values range from −40.4º to 21.69º, for both learning and test.

*Small to large* or *large to small* Ants may carry food items of different sizes on different traversals of the route, so we also test using small pitch variation on the stored images with large pitch variation on test and vice versa. The small and large distributions are as above. We do not include a difference in the mean of the variation, although it is possible that different size food items would also provide consistent bias in the pitch. We also do not represent the possibility that different food items might partially block the ants view in different ways.

For all five pitch variation possibilities, we test the directional error resulting from five alternative memory and visual processing assumptions:

(I) *Using spatially closest memory* Heading angles were recovered at every test location by computing the rIDF between current views and a memory 1 cm further along the route. Thus, images should be spatially well matched but are unlikely to be the same with regard to the pitch angle. Note this method makes the somewhat unlikely assumption that the ant knows where it is to within 1 cm and can recover the correct corresponding image from memory.
(II) *Using spatially near best matching memory* Simulated ants were allowed to search for the best rIDF score between current view and the visual memories taken at 1-cm intervals in the area 15 cm before and after the current location. Thus, they may potentially recover a more accurate heading direction by using a memory that has a more similar pitch but possibly greater displacement than 1 cm along the route. We omitted the memory at the current location to prevent perfect matching.
(III) *Using best matching memory across route* Simulated ants searched for the best rIDF score between current view and the visual memories taken at 1-cm intervals across the whole route (812 cm long). This is more consistent with current route following algorithms (Baddeley et al. 2012; Philippides et al. 2011) than the local search described above and does not require the ant to have indexed its memories by their location. Again, the memory of the current location was omitted.
(IV) *Disregard views at large pitch values* Ants may infer that they are viewing the world at a pitch angle not conducive to accurate heading recovery and opt to omit the view from memory, or not compute a new heading direction on test. This could improve matching but at the cost of potentially having gaps in the route in which correction does not occur. To simulate this selectivity, method III above was altered so that training images sampled at pitch greater than 10º or less than −10º (i.e. at the onset of visual compass degradation, Fig. 2c) were removed from the memory, and during test, any current image exceeding this pitch was ignored and the direction that was computed at the previous location selected instead.
(V) *Best matching memory of averaged images* Another means to overcome the influence of head pitch variation might be to store images not as instantaneous snapshots, but as averaged images taken over time. In this way, the stored view would contain information from a range of pitch angles and thus might more closely match the view from the same location regardless of pitch angle. ‘Average images’ were created every 1 cm by taking the mean of the current view and the previous four images separated at 1 cm. Method III was then used with these images as the memories to be compared to the current image.

### Effect on simulated navigation in the ant’s world

The above analysis will reveal the angular error caused by realistic pitch undulations at specific locations along a real ant route, and whether the error is reduced under certain processing assumptions. However, it is difficult to quantify whether the angular errors remaining would prevent an ant from retracing a learned path or not. In particular, the continuous nature of route following might offer robustness to instantaneous errors, by allowing correction in the next instant. We therefore tested the ability of a simulated ant to recapitulate the entire ant route in the simulated ant environment (see Fig. 4a for real ant route).

Images were again stored along the full ant route at 1 cm. The simulated ant would attempt to retrace the route from the starting point by first calculating the rIDF, for ±90º around the current heading, comparing against memories taken across the entire route (method III above). Once a global minimum was found, the ant would move 1 cm in that direction and the entire process was repeated until the ant reached home. In the case that the ant deviated from more than 1 m from the original route, or overshot the route length by 0.1 m (822 cm), the simulation was halted. The resulting path gives an indication of the robustness of navigation to disturbance of the visual input.

As above, we focus on five test scenarios: zero pitch training vs. zero pitch test as a control and all combinations of small and large pitch on both training and test, representing realistic situations of ants returning home with food of differing size.

## Results

### Do ants stabilise their head?

Homing ants were recorded with high-speed video as they ran over natural terrain. Five ants were selected for analysis as they carried a variety of objects that a homing ant is likely to possess while retracing her learned route: a small, medium or large piece of cookie (estimated mass and volume: 8 mg and 1 mm^3^; 16 mg and 2 mm^3^; 32 mg and 4 mm^3^ respectively); another ant [approximately 7.1–27 mg (Kühn-Bühlmann and Wehner 2006; Cerdá and Retana 1997)]; or nothing. Ants were highly motivated and thus moved quickly nestward but the differing weights of their bounty meant they moved at different speeds through the 4 cm recording area (see Fig. 1b). Figure 1b shows the time course of head and body pitch angles, relative to horizontal location of the neck joint in the camera image (i.e. location in their travel from left to right side of the frame), and Fig. 1a displays the distribution of pitch angles of the head for each ant. We stress that this is a simple observational study to support the parameters of head pitch variation used in our modelling, and not an experimental study to determine the causes of that variation.

We see that the body pitch is altered by the combination of uneven terrain and step cycle or leg configuration (it is not possible in our recordings to separate these factors, as unevenness of terrain in the depth axis of the camera obscured detail of footfall locations). If ants were actively stabilising, the head pitch should remain relatively constant, or at least fluctuate substantially less than the body pitch, but it is clear that for the most part it follows the body pitch. In some cases (ant with small cookie), the head movement is somewhat reduced relative to the body, but in other cases (ant with large cookie), it is increased. It also appears that carrying different items affects the median of the pitch as well as the distribution, but we would need more examples to conclude that this is a consistent effect. In every case, the ant experiences a wide range of pitches (at least 20–30 degrees) in this short time interval. There is also little consistency in the time/locations of higher or lower pitch from ant to ant as they encounter different small-scale undulations in the same terrain. We would consequently expect the same ant running repeatedly through the same part of a route, possibly carrying different food items, could be faced with the problem of matching images that are altered in pitch from one experience to the next by as much as ±30 degrees.

### The systematic effect of pitch on image difference

The lack of head stabilisation and consequent range of pitch experienced by an ant may not be a problem for the visual compass algorithm if this amount of pitch variation does not substantially affect ability to find the best directional match. In Fig. 2, we show the image difference function is affected by pitch, by comparing a reference image with no pitch to test images at pitches from −40 to 40 degrees. Eighty-one ant-eye images, taken every 10 cm along a real ant route, were generated in our reconstructed ant world (see ‘Methods’). For each, we calculated the rIDF as the viewing direction is rotated from 0° to 360° in 2° intervals. All rIDFs were then aligned so that the correct heading corresponded with 0°. As expected, at 0 degrees pitch of the test image, there is a perfect match at the correct direction (0°), surrounded by a monotonically increasing valley of image similarity. However, as a pitch difference between the test and reference view is introduced, the rIDF is clearly affected. The valley becomes much shallower as pitch difference increases, with additional local minima appearing at incorrect heading directions. Both factors would make a visual compass—which functions by selecting the minimum in the rIDF—less reliable. At each location, we also calculated the absolute difference between the selected direction (rIDF minimum) and the correct direction, and show the distribution of the errors as the pitch increases (Fig. 2b). Within the range of ±5° of pitch, the errors are relatively small (medians of 2° for both with 0° IQR for both), while at ±10° the median remains low (6° and 4° respectively) although the IQR increase significantly (92° and 114° respectively). For higher values of pitch mismatch, the median error increases substantially (e.g. 36° and 26° for ±15° respectively). Moreover, this assumes that the ant is able to detect a minimum (correct or incorrect), which should become increasingly difficult as the rIDF becomes flatter or noisier. As described in the methods, we take the ratio of the median rIDF to the minimum in the rIDF as an index of detectability; if this is near 1 then the directional information detectable in the rIDF is low. It is apparent in Fig. 2c that the detectability of the best match and thus the reliability or confidence in a directional choice also decreases substantially as the pitch difference is increased.

### Errors induced by pitch variation during route learning and recapitulation

The previous analysis compares the rIDFs for different pitches against a zero pitch reference image at the same location. However, a navigating ant would presumably experience a range of pitches both when learning and recapitulating a route. We examine whether a visual memory of a route that includes dense sampling for both location and pitch can be used to improve the directional choice made by the visual compass mechanism. As described in the methods, we sample pitch angles directly from those observed in real ants (either the ant carrying small cookie or ant carrying large cookie) and use this to vary visual input experienced along a route. We use zero pitch variation on learning and test as a control. In training, images are stored every 1 cm along the route, generating 812 memories for the test route to potentially exploit (see supplementary material for videos of training route for the various pitch conditions).

**Fig. 3:**
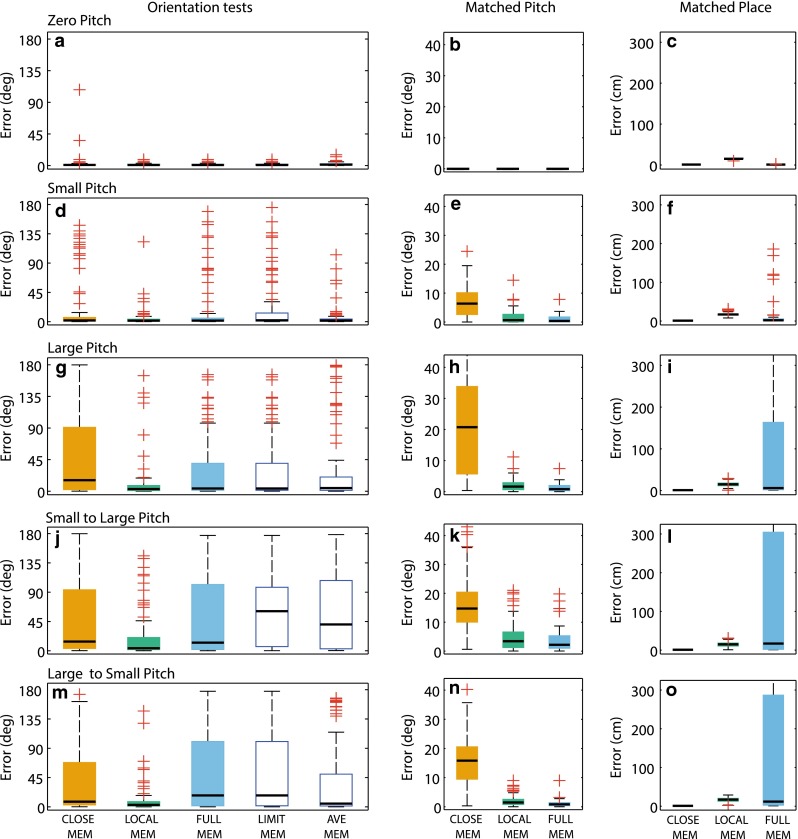
Approaches to reducing the impact of head pitch on visual compass. *Left column* (**a**, **d**, **g**, **j**, **m**) angular difference between the ant path and the direction selected at 81 test locations along a real ant route using five different memory and visual processing techniques: Closest Memory (*orange boxes*); Local Memory (*green boxes*); Full Memory (*blue boxes*); Limited Memory; and Average Image. Medians are shown by the *black bar* and the IQR by the *box*. From *top* to *bottom* are the combinations of pitch in memory and test: zero, small and large pitch in memory and test; followed by small pitch memory and large in test and vice versa. *Middle column* (**b**, **e**, **h**, **k**, **n**) angular error between the pitch angle at which the test image was sampled and the best match found in memory using the Closest, Local and Full Memory methods, respectively. *Right column* (**c**, **f**, **i**, **l**, **o**) distance in cm between the location at which the test image was sampled and the best match found in memory using the Closest, Local and Full Memory methods, respectively. Searching in local memories only allows a spatially close image to be found at a correlated pitch angle, hence good performance. However, when searching over the entire route memory, pitch matching dominates, leading to spatial mismatches and poor performance. Learning only at small pitch values <10° and averaging memory do not improve performance

We first assess the performance of the visual compass if the current view is compared with visual memory at the closest location (Fig. 3 box plots, orange filled boxes) and we use 1 cm further along the route to avoid the unrealistic accuracy that can occur if the exactly identical location is used. The data show the visual compass is accurate when tested with zero (Fig. 3a, median = 0.9º) or small pitch angles (Fig. 3d, m; medians = 1.9º and 7.9º, respectively). The worst performance is found when we test with large pitch regardless of the training condition (Fig. 3g, j; medians = 15.7º and 14.1º). Figure 3e, h, k, n shows that when using the nearest location, there can be significant mismatch in pitch, which in turn leads to the increased angular errors.

We then assessed performance when the visual compass is allowed to find the best matching image in the 30 training images closest to the current location, that is, 15 steps before and after the test location. In this case, the simulated ant may find a good match with the current image in terms of pitch while being constrained to only small spatial mismatches. The data (Fig. 3, green box plots) show that the visual compass combined with a local search through dense memories remains accurate across all memory and test scenarios (Fig. 3a, d, g, j, m; medians = 0.8º, 1.5º, 3.1º, 3.9º, 3.0º respectively). Figure 3e, h, k, n shows that by searching through nearby memories sampled across a range of pitch angles, the simulated ant can find an image stored at a similar pitch as the test image, and thus a better match than for the spatially closest image.

While the above data shows that dense memories can provide robustness to pitch disturbances, both methods assume that ants have indexed their memories by location and can recover the relevant memory (within 1 cm) or set of memories (within 15 cm). Behavioural data suggests ants can follow routes from arbitrary points along them, i.e. in ignorance of their spatial location (Kohler and Wehner 2005; Mangan and Webb 2012; Narendra 2007a, b), and recent algorithms have replicated this capability by assuming ants use the visual compass to find the best matching memory across the entire route (Baddeley et al. 2012). We therefore assessed whether finding the best matching image across the entire 812 route memories (excluding the current location) was also robust against pitch variation. We find that performance using all route memories remains statistically indistinguishable from the local search except when large pitch is present in the memory (*p* < 0.02 using Wilcoxon rank-sum test) (Fig. 3a, d, g, j, m blue box plots, medians = 0.8º, 1.6º, 3.9º, 12.5º, 17.6º). The reason for the increase in angular error with a global search is clear when the position and pitch of the matched images are examined (Fig. 3h, i, n, o). When we allowed to search the entire route memory, best matches are found at similar pitch angles to that on the test, but this often leads to image matches with images far from the test location (upper IQR around 3 m from the test site). This aliasing of views leads to erroneous heading directions.

It is clear that most degradation in performance occurs when there are large pitch disturbances on the training or test set. Thus, if ants were able to detect the angle of pitch (see discussion), they may actively chose to omit learning views, or updating their home direction, until the head returns to a more suitable position. However, such a schema might be costly as it may produce gaps in the memory or lead to errors at key points when later navigating. We report that this method indeed leads to decreased performance when compared to perfect memory when trained with small pitch and tested with large pitch (Fig. 3j; medians = 60.8º, *p* < 0.01 Wilcoxon rank-sum test). Results in other conditions are similar to the full memory condition (Fig. 3 a, d, g, m; medians = 0.8º, 2.1, 3.9º and 17.6º, respectively). In summary, this approach does not improve performance.

Finally, we tested the performance of the visual compass when memories were converted from single snapshots to averaged images. That is, each stored memory is the mean of the last five steps and should therefore incorporate various head pitch angles. We test the heading error when comparing the current view with the averaged views stored across the entire route. We find that this also does not improve performance over the perfect memory of instantaneous images (Fig. 3a, d, g, j, m; medians = 1.3º, 1.5º, 4.5º, 40.4º, 4.9º).

### The effect of error on route following

*C. velox* ants experience considerable variation in head pitch when running over moderate terrain (Fig. 1), and this could induce substantial error in the directional information of a visual compass (Fig. 2). Large angular errors remain when matching memories across the entire route (Fig. 3, blue box plot). However, as route following with a visual compass involves successive re-orientations to the best matching direction, we would expect it to be somewhat robust against small or occasional directional errors. That is, if the ant deviates from the learned path due to an erroneous visual compass reading, it might still recover the route in the next step through a better match, and hence reach home.

To evaluate the error tolerance of route following with a visual compass, we simulated an ant driven by visual compass with perfect memory (at 1-cm intervals) to travel along our reconstructed real ant route, using either zero, small or large pitch variation as described above. For route recapitulation, the simulated ant scans ±90 degrees from the current heading direction, selecting the view with the lowest image difference from the entire bank of stored memories. The corresponding heading is then chosen for the next step. If the simulation deviates more than 1 m from the original ant route, or exceeds the route length by 0.1 m, it is stopped. These conditions mirror actual behaviour of the animal as substantial deviations from the route corridor are seldom observed and nest bound ant routes are not tortuous (Mangan and Webb 2012).

**Fig. 4:**
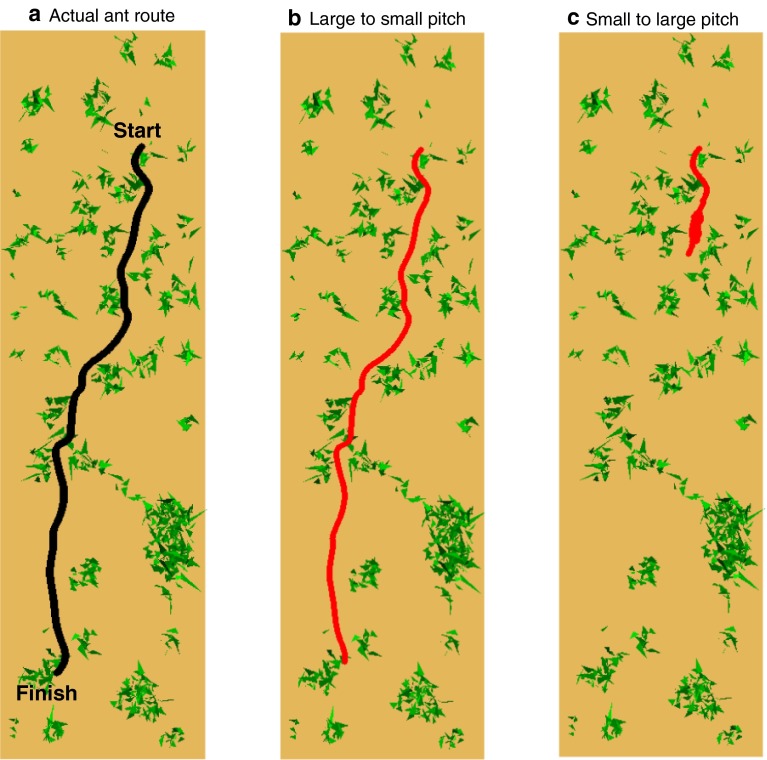
Navigating real ant routes with realistic head movement. **a** Actual route followed by an ant through its cluttered environment in Sevilla, Spain, acting as ground truth. **b**, **c** Example paths followed by simulated ant following visual compass methodology and realistic head pitch for memory and route recapitulation (**b** large to small; **c** small to large). The iterative computation of direction can compensate for the errors introduced by pitch (**b**) but is still susceptible to failure (continuous loops in this case) (**c**)

Under the conditions where the training and test pitch were drawn from the same distribution (i.e. zero, small and large pitch), the entire route could be followed with no errors despite the difference in the visual input (data not shown). Where the pitch angle varies (e.g. Small to Large and vice versa), errors were often encountered. However, the capacity to correct over successive steps appears to offer some robustness as shown in Fig. 4b, but does not offer complete robustness as demonstrated by the catastrophic error in Fig. 4c. In contrast, ants returning home rarely deviate from their path, even in the absence of path integration (Kohler and Wehner 2005; Mangan and Webb 2012; Wystrach et al. 2011) and thus may have developed strategies to deal with disturbances in visual input.

## Discussion

The study of visual navigation in ants has led to the development of a number of algorithms that reproduce aspects of their behaviour (Cartwright and Collett 1982; Hafner 2001; Möller 2001; Stürzl and Mallot 2006; Wystrach et al. 2013; Zeil et al. 2003), including the ability to follow extended visual routes (Baddeley et al. 2011, 2012). In general, these models use some form of retinotopic image matching to recover a heading direction. It is increasingly argued that ants use essentially raw or whole image matching, with minimal image processing and no feature extraction. This means the potential distortions introduced by variability in head pitch or roll could have a significant effect, but most models assume stable visual input.

However, the evidence for head stabilisation is not clear. Ants running on flat ground, such as the Tunisian salt pans, may experience only small disturbances, although even here, as discussed in the introduction, some variation in head pitch during the step cycle is observed (Steck et al. 2010). Ants experiencing gradual or consistent change in body orientation due to a substrate slope show some ability to compensate their head position, but they do not fully compensate for substantial slopes (Wohlgemuth et al. 2002; Weihmann and Blickhan 2009). High-speed video provides the opportunity to examine whether there is rapid compensation for the fine-scale variation in body position caused by walking over moderate variations in terrain. Our results suggest that, at least for *C. velox* head pitch, no such compensation occurs. Rather, head position varies with body position as determined by the terrain combined with the gait and is also influenced by any object that the ant might be carrying. Consequently, we report rapidly varying head pitch: up to ±30 degrees. We note here that preliminary processing of high-speed video of a *C. fortis* ant running on flat terrain (supplementary data from Steck et al. 2010) also reveals substantial variation in head pitch (at least ±10 degrees) (see supplementary material). Experiments inducing roll in the body orientation of ants also show some, but not full, compensatory roll of their head (Raderschall et al. 2014) and ants under these conditions can be observed to experience a similar range of roll (i.e. ± 15 degrees) over a short timescale (C. Raderschall, personal communication). Note that this suggests our analyses of the effects of head motion on navigation are conservative, as roll would provide additional distortion.

We use simulation of the ant’s visual input, based on a reconstruction of a real ant environment, to assess the effects of the observed variation in pitch on current navigational methods. It is clear that pitch differences have a significant effect on image matching algorithms such as the visual compass. For pitch differences of 15 degrees or more, the minimum in the rIDF is hard to detect and often falls in the wrong location, which would result in errors in directional choice. The accuracy of visual compass can be improved if ants have a dense memory of views around its current vicinity containing a large range of pitches similar to the pitch value current experienced. The ant would then potentially have available a ‘nearby’ memory at the same pitch as the current view and therefore be able to more accurately recover the correct direction than using nearest memory, which might be at the wrong pitch. However, such a local search in memories would require memory indexing which does not fit with behavioural data (Kohler and Wehner 2005; Mangan and Webb 2012; Narendra 2007a, b). When tested with visual memories with large pitch from the entire route, performance degrades because pitch matching is favoured at the expense of proximity. We then tested in a full simulation of route following, to quantify the impact of instantaneous errors on a real navigation task. We conclude that while the iterative process of route following offers some tolerance to pitch induced heading errors, the variability of head pitch observed in ants is potentially problematic for the visual compass hypothesis, at least in its most straightforward form, and in our challenging environment comprising a dense array of proximal grass tussocks without any distal cues.

The ant visual system (eye and optic lobe) filters information directly reducing the computational load on the brain. We therefore investigated whether rudimentary visual averaging might restore visual matching without increasing memory load. Each image should encompass a range of pitches, and this may be more robust for image matching than the single snapshots used in current methods. However, we find the results were no better than for simple image memory.

Ants might be able to detect when their heads are at a level pitch and roll, or intermittently adopt a stereotyped pose with a levelled head, and only ‘take snapshots’ at these key moments. It is plausible that the ant has sensory mechanisms that could enable it to detect the pitch and roll of its head, for example through proprioception relative to gravity (Seidl and Wehner 2008), or visual information (possibly from the ocelli) about the horizon [as in locusts (Taylor 1981)]. However, using knowledge of the pitch to reject memories, and prevent comparisons, at pitches of more than 10 degrees did not significantly improve the performance in our tests.

Ants could also potentially use pitch information directly to make the required correction to the image, i.e. to perform mental rotation. Another possibility is that the mechanisms for visual navigation are not so strongly retinotopic as image matching algorithms suggest. Models of navigation that use landmark bearings may be less affected by pitch and roll, which principally distort height (Collett 1992). Yet such methods are known be ineffective in natural environments where landmarks can be hard to identify and do not fit well with behavioural studies of visual homing in insects (Mangan and Webb 2009).

A possibility is that other view matching strategies could reduce or compensate for such inaccuracies. The visual compass approach used here does not capture all aspects of ant visual guidance. For example, ants recapitulate routes without scanning at each step, and recover routes after a deviation (Collett 2010; Wystrach et al. 2012) suggestive of alternative visual strategies. Finally, sky compass may also be helpful. It is important to note, however, that variability in pitch and roll to the extent described here could also affect the sky compass. That is, the input to polarisation sensitive ommatidia in the dorsal rim of the ant eye depends on their orientation relative to the sky pattern of polarisation which in general is not a uniform across the entire sky; hence, pitch and roll of the head will alter the input. Again, it is possible that subsequent neural filtering could reduce the impact of this variation or that active correction from detection of the head position is used to compensate.

As computational models are developed that can account for increasingly complex behaviours, it is vitally important to introduce realistic constraints derived from animal observation. Here, we have challenged the assumption that ants stabilise their heads and introduced the constraint that navigation must be robust to pitch variation. Only through repeatedly and rigorously challenging assumptions can we hope to refine our hypotheses to reveal the secrets of these amazing navigators. ‘By entertaining this kind of bottom–up approach to understanding the organization of behaviour, we might finally follow the routes originally taken by evolution in orchestrating the different guiding mechanisms and knitting them into what Fabre (1882) called “the insect’s awe-inspiring system of navigation”’ (Wehner et al. 1996).

## Electronic supplementary material

Supplementary material 1 (WMV 3398 kb)

Supplementary material 2 (WMV 5330 kb)

Supplementary material 3 (WMV 1508 kb)

Supplementary material 4 (WMV 2242 kb)

Supplementary material 5 (WMV 708 kb)

Supplementary material 6 (EPS 9 kb)

Supplementary material 7 (AVI 1104 kb)

Supplementary material 8 (AVI 1083 kb)

Supplementary material 9 (AVI 1091 kb)

## Abbreviations

IQR: Interquartile range. The difference between the 75 and 25 % percentiles of the data
rIDF: Rotational image difference function. The resulting function produced after computing the image difference between a reference image and the current view across a defined rotational range
